# Unfolding the genetic map of monogenic liver diseases in Egypt

**DOI:** 10.1007/s00439-025-02776-4

**Published:** 2025-10-30

**Authors:** Hanaa El-Karaksy, Engy A. Mogahed, Sherif Baroudy, Haytham Ghita, Afaf Enayet, Marwa El-Sharkawy, Noha A. Radwan, Heba Hosny, Mohamed A. Elmonem

**Affiliations:** 1 Department of Pediatrics, Faculty of Medicine, Cairo University, Cairo, Egypt; 2 Department of Clinical and Chemical Pathology, Faculty of Medicine, Cairo University, Cairo, Egypt; 3 Fellow of Medical and Clinical Genetics, National Institute of Neuro-Motor System, El-Tahrir City, Egypt

## Abstract

Monogenic liver disorders constitute a major disease burden in pediatric patients presenting with manifestations of liver disease. This is particularly significant in countries with high rates of consanguinity as in Egypt. We recruited 228 infants and children suffering from various forms of liver disease with suspected monogenic background and no conclusive biochemical diagnosis. They presented to the Pediatric Hepatology Unit at Cairo-University-Children’s-Hospital, Egypt, over the period April 2017-June 2023 and were referred for genetic diagnosis by various next-generation-sequencing technologies. One-hundred and eighty-five children (81.1%) had a significant genetic finding. Those included 88 children with a main presentation of organomegaly, 83 with cholestasis, 8 with acute liver failure and 6 with hyperbilirubinemic syndromes. One-hundred seventy-five disease-causing variants (51 pathogenic, 86 likely-pathogenic and 38 of uncertain-significance) in 72 different genes were detected in our cohort, including 85 novel variants in 49 different genes. Variants in *AGL* (Glycogen storage disease type-III; GSD3), *ABCB4* (Progressive familial intrahepatic cholestasis type-III; PFIC3) and *ABCB11* (PFIC2) genes were the most common affecting 19, 14 and 13 children, respectively. This study is the first to provide the landscape of pediatric monogenic liver diseases in a homogenous population from the Middle East and Africa, an area notoriously known to be poorly represented in genetic database and literature. Furthermore, our presented data are extremely important for genetic counseling and prenatal diagnosis and will further guide national health care policy makers to prevent or mitigate the effects of such disorders in the future.

**Supplementary Information:**

The online version contains supplementary material available at 10.1007/s00439-025-02776-4.

## Introduction

In the literature, the genetic basis of rare diseases is overwhelmingly represented from developed nations in Europe, East Asia and North America with minimal representation from Africa or the Middle-East and monogenic liver diseases are no exception. Chronic liver diseases in infancy and childhood are commonly caused by genetic alterations of metabolic pathway genes, which when defective affect the liver usually among other organs (Stalke et al. 2018). Monogenic liver disorders often present with overlapping phenotypes and the majority lack definitive laboratory or histological markers, thus in the absence of genetic tools, this may lead to prolonged diagnostic uncertainty and a chance of faulty treatment (Fang et al. 2021).

Currently, the most commonly used genetic testing techniques include targeted gene panels, clinical exome sequencing and whole exome sequencing. Genetic panels are focused mainly on a limited number of suspected diseases, and it has better coverage of the genes and is of low cost. Exome sequencing is particularly recommended to improve the diagnostic yield in cases of an uncertain phenotype or unclear clinical scenarios (Kingsmore and Saunders 2011; Nicastro and D’Antiga 2018). A tight liaison between the laboratory geneticist and the clinician is necessary to ascertain the correct diagnosis that involves filtering of gene candidates, on the basis of phenotypic features (Rabbani et al. 2012).

Over the past few years, several studies have reported the genetic spectrum of monogenic liver diseases in multiple developed countries (Stalke et al. 2018; Fang et al. 2021; Kulecka et al. 2017; Lipiński et al. 2020; Hegarty et al. 2021; Pelusi et al. 2021; Kong et al. 2022; Almes et al. 2022). The diagnostic yield in these cohorts ranged between 7 and 68% depending on the design of the study, number of patients and clinical groups of liver diseases targeted.

To the best of our knowledge, this is the first study mapping the clinical and genetic spectra of a large pediatric cohort presenting with a diverse group of monogenic liver disorders in Egypt, the Middle-East and Africa. Although some studies, particularly from Saudi Arabia, reported the role of genetic testing in identifying causes of pediatric acute liver failure (Alhadab et al. 2023) or infantile cholestasis (Al-Hussaini et al. 2024), this is the first study to investigate the full spectrum of pediatric monogenic liver diseases in a homogenous population in the region.

## Patients and methods

### Patients

This study included infants and children suffering from various forms of liver disease with suspected genetic background. The data of 228 unrelated pediatric patients with the clinical suspicion of a monogenic liver disease presenting to the Pediatric Hepatology Unit at Cairo University Children’s Hospital over a 6-year period from April 2017 to June 2023, were retrospectively obtained.

The study included all infants and children of both genders aged from birth till 18 years in which a biochemical diagnosis that fully explained their hepatic phenotypes could not be obtained. Recruited patients were either presenting with neonatal cholestasis, organomegaly, acute liver failure (neonatal or later in life) or hyperbilirubinemic syndromes (conjugated or unconjugated). Neonatal cholestasis was defined as direct hyperbilirubinemia (> 1 mg/dl) with an onset in the neonatal period (cholestatic patients who had neonatal liver cell failure were included in the acute liver failure group). Acute liver failure was defined as presence of biochemical liver injury with absence of history of chronic liver disease and coagulopathy (INR > 1.5) not corrected by vitamin K associated with hepatic encephalopathy or INR > 2 not corrected by vitamin K with or without encephalopathy. The organomegaly group included patients with hepatomegaly, splenomegaly or hepatosplenomegaly who did not have neonatal cholestasis or acute liver failure. Patients with hyperbilirubinemic syndromes included those with direct or indirect hyperbilirubinemia with otherwise free clinical examination and normal liver function tests.

Positive consanguinity and previous reported cases in the family were strong suspicion points for monogenic liver diseases; however, they were not an indication for recruitment. Patients were subjected to genetic testing after exclusion of other causes of liver diseases, such as biliary atresia, infections or autoimmunity and only after performing biochemical metabolic investigations that may detect a metabolic genetic liver disease also revealing no diagnosis. All subjects with available results for genetic testing (target gene panels, exome-sequencing or genome-sequencing) whether positive or negative were included.

The study was conducted in accordance with the declaration of Helsinki for studies involving human participants and was approved by the institutional research ethics committees at Faculty of Medicine, Cairo University (Approval code: #N-16-2022). Written informed consents were obtained from parents/legal guardians of all participants.

### Methods

According to clinical needs different sequencing approaches were used for our cohort. Whole exome-sequencing was performed for 123 children, while for 80 patients a panel of 206 metabolic genes was investigated (CentoMetabolic, Centogene, Rostock, Germany). For another 22 children clinical exome (≈ 6600 genes who are known to cause human disease) was evaluated, while whole genome-sequencing was conducted for only 3 children. EDTA blood samples were obtained from all clinically suspected individuals. Genomic DNA was enzymatically fragmented, and regions of interest were enriched using DNA capture probes. The coding regions plus at least 20 bp of flanking intronic sequences and known pathogenic/likely pathogenic variants (coding and non-coding) were targeted for analysis. The final indexed libraries were sequenced on next generation sequencing platform NovSaq(Illumina, San Diego, CA, USA).

Data analysis, including alignment to the hg19 human reference genome (Genome Reference Consortium GRCh37), variant calling, and annotation was performed at Centogene Inc, Rostock, Germany. All variant nomenclature complies with the guidelines of the human genome variation society (HGVS) (den Dunnen et al. 2016). All disease-causing variants previously reported in HGMD^®^ (https://www.hgmd.cf.ac.uk), in ClinVar (https://www.ncbi.nlm.nih.gov/clinvar/) or in the literature were prioritized and all pathogenic/likely pathogenic variants according to the American College of Medical Genetics (ACMG) criteria for the pathogenicity of genetic variants were reported (Richards et al. 2015). Variants of uncertain significance (VUS) according to the ACMG criteria were only reported whenever no relevant pathogenic/likely pathogenic variants have been identified, if only matching the patient phenotypes perfectly and when evidence strongly suggests their pathogenicity, such as being absent or extremely rare and absent in homozygosity in population database, lying within an important protein domain or a mutational hotspot of the gene, strongly predicted to be deleterious by most predictor software or having a splicing effect and/or previously reported in the literature, HGMD or ClinVar as disease causing for the patient phenotype. Copy number variants (CNVs) were also evaluated whenever a conclusive diagnosis was not reached with small variants.

## Results

### Demographic data of Egyptian children with monogenic liver diseases

The study included 228 unrelated pediatric patients (139 males) who were suspected to have a monogenic liver disease over the study period. Median (IQR) age at the time of genetic testing was 32 (10–78) months. Next-generation sequencing techniques were conducted for the probands in order to reach a genetic diagnosis according to their phenotypes. Reverse clinical phenotyping was performed for all received NGS results. If the reported diagnostic variant was deemed unsuitable for the child’s phenotype, particularly in the case of VUS variants, the patient was considered to have no significant genetic diagnosis.

One-hundred and eighty-five children (81% of the recruited cohort, 115 males, 29 (9.5–75.5) months) had a significant genetic finding. Those included 88 children with a main presentation of organomegaly, 83 with cholestasis, 8 with acute liver failure and 6 with hyperbillirubinemic syndromes. Consanguinity was positive in 87.2% of families; while 36.5% reported history of previously affected siblings. There was no significant difference between the genetically-confirmed and children without significant findings regarding age, sex, age at genetic testing, consanguinity status and positive family history. Suspected children with hyperbilirubinemia had a lower diagnostic yield compared to other phenotypes, *P* = 0.001 (Table 1).

**Table 1:** Demographic and clinical data of recruited patients (*N*=228)

	All recruited children (*N*=228)	Genetically confirmed children (*N*=185)	Children with no genetic diagnosis (*N*=43)	*P*-value*
**Demographic data**				
Age in months at genetic testing: median (IQR)	32 (10-78)	29 (9.5-75.5)	35 (11-89)	0.440
Sex (Male/Female)	139/89	115/70	24/19	0.725
Consanguinity (Positive/Negative)	199/29	163/22	36/7	0.912
Family history (Positive/Negative)	64/164	50/135	14/29	0.340
**Clinical phenotypes**				
Organomegaly; number (%)	102 (44.7%)	88 (47.6%)	14 (32.6%)	0.247
Cholestasis; number (%)	101 (44.3%)	83 (44.9%)	15 (36.6%)	0.273
Liver cell failure; number (%)	12 (5.3%)	8 (4.3%)	4 (9.8%)	0.156
Hyperbilirubinemia; number (%)	13 (5.7%)	6 (3.2%)	7 (17%)	0.001
**Diagnostic yield (Total)**	185/228 (81.1%)			
**According to sequencing approach**				
Whole exome	98/123 (79.7%)			
Metabolic genetic panel (206 genes)	67/80 (83.7%)			
Clinical exome (6600 genes)	18/22 (81.8%)			
Whole genome	2/3 (66.7%)			
**According to phenotype**				
Organomegaly	88/102 (86.3%)			
Cholestasis	83/101 (82.2%)			
Liver cell failure	8/12 (66.7%)			
Hyperbilirubinemia	6/13 (46.2%)			

### Genotypic spectrum of Egyptian children with monogenic liver diseases

**Fig. 1:**
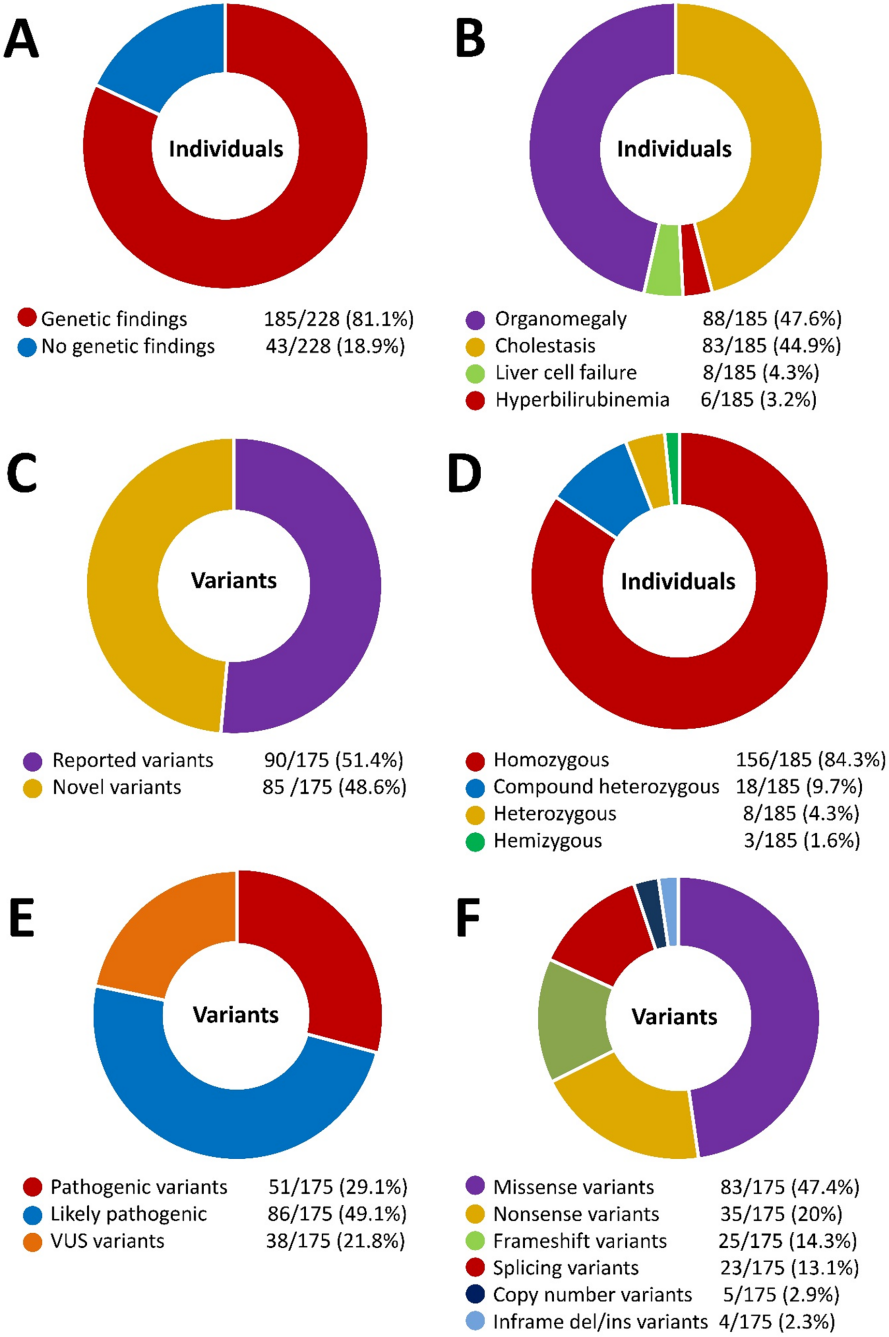
A representation of the distribution of recruited individuals over different disease and variant categories. **A** Individuals with genetic findings versus no genetic findings. **B** Individuals with positive finding distribution over the four main presenting phenotypes. **C** Previously reported versus novel variants. **D** Patterns of inheritance of diagnosed probands. **E** Level of pathogenicity of reported variants. **F** Different types of variants detected in our cohort according to their protein impact

One hundred and seventy-five potentially disease-causing variants belonging to 72 different genes affecting the liver were detected in our cohort, including 85 novel variants (48.6%) and 90 (51.4%) previously reported (Supplementary Table 1). Of the genetically confirmed children 94% had autosomal recessive disorders (84.3% with homozygous variants and 9.7% with compound heterozygous variants). Autosomal dominant disorders represented only 4.3% of diagnosed cases, while 1.6% had X-linked disorders (Fig. 1). According to the ACMG criteria for the evaluation of pathogenic variants (Richards et al. 2015), 29.1% of detected disease-causing variants were evaluated as pathogenic, 49.1% as likely-pathogenic and 21.8% as variants of uncertain significance (VUS). When variants were categorized according to their impact, missense variants were the dominant form constituting 47.4% of variants. Other types of variants included stop-gain variants (20%), frameshift variants (14.3%), splicing variants (13.1%), copy number variants (2.9%) and in-frame indels (2.3%).

**Fig. 2:**
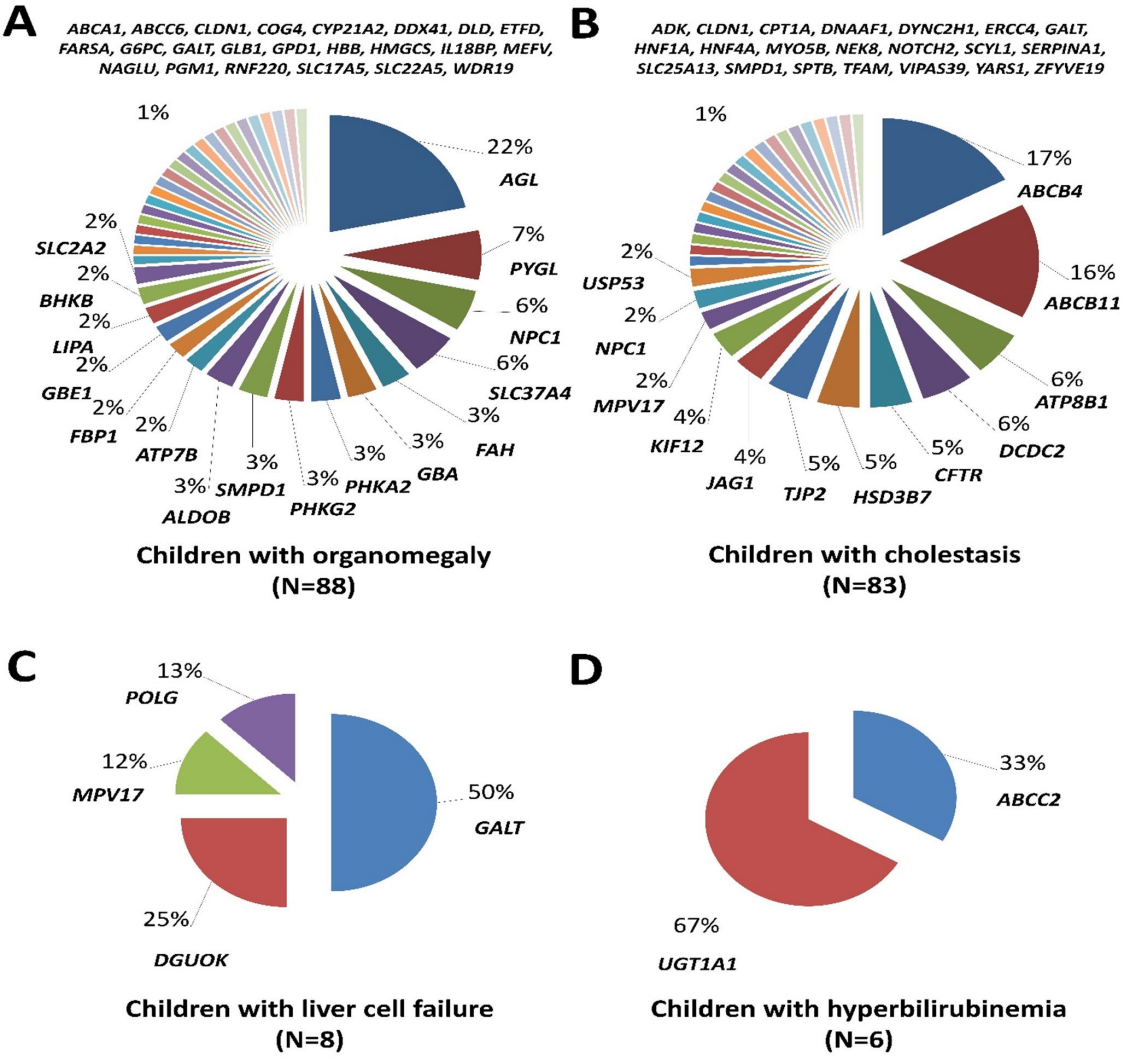
Frequency of detected genes responsible for the main monogenic liver disease phenotypes in Egypt. **A** Organomegly. **B** Cholestasis. **C** Liver cell failure. **D** Hyperbilirubinemia. Genes with one family each are named above the pie charts

Concerning the different phenotypic groups, 82 disease causing variants belonging to 40 different genes were detected in children who presented primarily with organomegaly (Table 2). *AGL* gene causing glycogen storage disease IIIa/IIIb (GSD3a/3b) was the most common gene affecting this group (19 children/14 variants). This was followed by *PYGL* gene (GSD6, 6 patients/7 variants), *NPC1* (Niemann-Pick disease type C1, 5 patients/6 variants) and *SLC37A4* (GSD1b/1c, 5 patients/4 variants) (Fig. 2A). Overall, GSD collectively constituted 39/88 of organomegaly patients (44.3%) and 32/87 of detected variants (36.8%). The most common detected variant in this group was the exon 28–30 deletion in the *AGL* gene detected in five unrelated Egyptian families responsible for approximately 25% of affected families with this gene, which may indicate a founder effect for this variant in Egypt. However, all other genes expressed high levels of heterogeneity as all other detected variants were either present in one or two families each.

Regarding the genetically confirmed cases presenting with cholestasis, 78 disease causing variants belonging to 34 different genes were detected (Table 3). *ABCB4* gene causing progressive familial intrahepatic cholestasis type 3 (PFIC3) with high gamma-glutamyl transferase (GGT) was the most common gene (14 children/13 variants). It was very closely followed by *ABCB11* gene causing PFIC2 with normal GGT (13 patients/12 variants), then by *ATP8B1* (PFIC1, 5 patients/5 variants) and *DCDC2* (neonatal sclerosing cholangitis, 5 patients/4 variants) (Fig. 2B). Overall, eight different PFIC syndromes collectively constituted 43/83 cholestatic patients (51.8%) and 42/78 of detected variants (53.8%). The most common detected variant in the cholestasis group in our study was the splice site variant NM_025193.3:c.694 + 2T > G in the *HSD3B7* gene causing bile acid synthesis defect type 1 detected in three unrelated Egyptian families and seems to have a founder effect in Egypt as the only pathogenic variant detected in this gene.

As for the eight genetically confirmed children presenting with acute liver-cell failure they were suffering from galactosemia (*GALT* gene, 4 patients/3 variants) and three different types of mitochondrial depletion syndromes (MTDPS): *DGUOK* gene (MTDPS3, 2 patients/2 variants), *MPV17* (MTDPS6, 1 patient/1 variant) and *POLG* (MTDPS4, 1 patient/2 variants) (Table 4; Fig. 2C).

Finally, only six children were genetically confirmed with genes causing hyperbilirubinemia syndromes. Five variants in *UGT1A1* gene causing Crigler-Najjar syndrome, type I/II were detected in four unrelated patients (3 homozygous and 1 compound-heterozygous), while two unrelated patients were reported with two disease causing variants in the *ABCC2* gene causing Dubin-Johnson syndrome (Table 5; Fig. 2D). Supplementary Table 2 provides the global allelic frequencies of all detected variants in our cohort in gnomAD population database (version v.4.1.0) https://gnomad.broadinstitute.org/ and details of their pathogenicity scoring according to the ACMG criteria (Richards et al. 2015; Tavtigian et al. 2020).

Notably, several genes were represented in different phenotypic groups, such as the *GALT* gene (galactosemia) reported as presenting with acute liver failure in 4 families, cholestasis (1 family) and organomegaly (1 family), *NPC1* (Niemann-Pick C1) reported with organomegaly (5 families) and cholestasis (2 families), *SMPD1* (Niemann-Pick A/B) reported with organomegaly (3 families) and cholestasis (1 family) and *MPV17* (MTDPS6) reported with a main presentation of cholestasis (2 families) and liver cell failure (1 family). This further emphasizes the complexity of phenotypic heterogeneity of many of the monogenic liver diseases diagnosed in our cohort.

**Table 2:** Variants detected in Egyptian children with a primary presentation of organomegaly (*N*=88)

Gene	Disease	Inh	Patients	HGVSC	HGVSP	Variant type	Zygosity	ACMG
***ABCA1***	Tangier disease	AR	1	NM_005502.4:c.5317G > A#	p.(Val1773Met)	Missense	Homo	VUS
***ABCB4***	Cholestasis, progressive familial intrahepatic 3	AR	2	NM_000443.4:c.1745G > A	p.(Arg582Gln)	Missense	Homo	LP
***ABCC6***	Pseudoxanthoma elasticum	AR	3	NM_001171.6:c.3823C > T	p.(Arg1275*)	Nonsense	Homo	P
***AGL***	Glycogen storage disease IIIa/IIIb	AR	4,5,6,7,8	chr1:99910707-99913753del	Exon 28–30 deletion	CNV deletion	Homo	P
			9,10	NM_000642.3:c.2590C > T	p.(Arg864*)	Nonsense	Homo	P
			11,12	NM_000642.3:c.1589C > G	p.(Ser530*)	Nonsense	Homo	P
			13,14	NM_000642.3:c.1859T > C	p.(Leu620Pro)	Missense	Homo	P
			15	NM_000642.3:c.2488G > T#	p.(Glu830*)	Nonsense	Homo	LP
			16	NM_000642.3:c.3980G > A	p.(Trp1327*)	Nonsense	Homo	P
			17	NM_000642.3:c.3816_3817del	p.(Gly1273Asnfs*18)	Frameshift	Homo	P
			18	NM_000642.3:c.3052_3053del#	p.(Leu1018Serfs*51)	Frameshift	Homo	LP
			19	NM_000642.3:c.1859T > C	p.(Leu620Pro)	Missense	Double-Hetero	P
				NM_000642.3:c.256C > T	p.(Gln86*)	Nonsense		LP
			20	NM_000642.3:c.2179_2218dup#	p.(His740Argfs*12)	Frameshift	Homo	LP
			21	NM_000642.3:c.1859T > C	p.(Leu620Pro)	Missense	Double-Hetero	P
				NM_000642.3:c.3980G > A	p.(Trp1327*)	Nonsense		P
			22	NM_000642.3:c.256C > T	p.(Gln86*)	Nonsense	Homo	P
***ALDOB***	Fructose intolerance, hereditary	AR	23	NM_000035.4:c.799 + 1G > A		Splice donor	Homo	P
			24	NM_000035.4:c.10C > T	p.(Arg4*)	Nonsense	Homo	P
***ATP7B***	Wilson disease	AR	25	NM_000053.4:c.3904-2A > G		splice acceptor	Homo	P
			26	NM_000053.4:c.1285 + 5G > T		splice donor	Double-Hetero	P
				NM_000053.4:c.3275C > T	p.(Thr1092Met)	Missense		LP
***CLDN1***	Ichthyosis, leukocyte vacuoles, alopecia, and sclerosing cholangitis	AR	27	NM_021101.5:c.175A > G#	p.(Thr59Ala)	Missense	Homo	VUS
***COG4***	Congenital disorder of glycosylation, type II	AR	28	NM_015386.3:c.1798G > A#	p.(Val600Met)	Missense	Homo	VUS
***CYP21A2***	Adrenal hyperplasia, congenital, due to 21-hydroxylase deficiency	AR	29	NM_001368143.2:c.-126C > G	p.(Val282Leu)	Splice acceptor	Double-Hetero	LP
				NM_001368143.2:c.844G > T		Missense		P
***DDX41***	Myeloproliferative/lymphoproliferative neoplasms, familial	AD	30	NM_016222.4:c.1534G > T#	p.(Glu512*)	Nonsense	Homo	LP
***DLD***	Dihydrolipoamide dehydrogenase deficiency	AR	31	NM_000108.5:c.685G > T	p.(Gly229Cys)	Missense	Homo	P
***ETFDH***	Glutaric acidemia IIC	AR	32	NM_004453.4:c.807A > C	p.(Gln269His)	Missense	Homo	P
***FAH***	Tyrosinemia, type I	AR	33	NM_000137.4:c.709C > T	p.(Arg237*)	Nonsense	Homo	P
			34	NM_000137.4:c.554-1G > T		Splice acceptor	Homo	P
			35	NM_000137.4:c.497T > G	p.(Val166Gly)	Missense	Double-Hetero	LP
				NM_000137.4:c.192 + 1G > A#		Splice donor		LP
***FARSA***	Rajab interstitial lung disease with brain calcifications 2	AR	36	NM_004461.3:c.1210C > T	p.(Arg404Cys)	Missense	Double-Hetero	VUS
				NM_004461.3:c.1297G > A#	p.(Gly433Arg)	Missense		VUS
***FBP1***	Fructose-1,6-bisphosphatase deficiency	AR	37	chr9:93,639,344-94639132del		CNV del	Homo	LP
			38	NM_000507.4:c.386G > T#	p.(Cys129Phe)	Missense	Homo	LP
***G6PC***	Glycogen storage disease Ia	AR	39	NM_000151.4:c.247C > T	p.(Arg83Cys)	Missense	Homo	LP
***GALT***	Galactosemia	AR	40	NM_000155.4:c.404C > T	p.(Ser135Leu)	Missense	Homo	LP
***GBA***	Gaucher disease	AR	41,42	NM_000157.4:c.1448T > C	p.(Leu483Pro)	Missense	Homo	P
			43	NM_000157.4:c.1241T > G#	p.(Val414Gly)	Missense	Homo	LP
***GBE1***	Glycogen storage disease IV	AR	44,45	NM_000158.4:c.476C > T#	p.(Pro159Leu)	Missense	Homo	VUS
***GLB1***	GM1-gangliosidosi	AR	46	NM_000404.4:c.569_572del	p.(Lys190Serfs*27)	Frameshift	Homo	P
***GPD1***	Hypertriglyceridemia, transient infantile	AR	47	NM_005276.4:c.806G > A	p.(Arg269Gln)	Missense	Homo	LP
***HBB***	Thalassemia, beta	AR	48	NM_000518.5: c.92 + 6T > C		Splice donor	Homo	P
***HMGCS2***	HMG-CoA synthase-2 deficiency	AR	49	NM_005518.4:c.1118A > T#	p.(Tyr373Phe)	Missense	Homo	VUS
***IL18BP***	Hepatitis, fulminant viral, susceptibility to	AR	50	NM_001039660.2:c.15_16del#	p.(His5Glnfs*40)	Frameshift	Homo	VUS
***LIPA***	Wolman disease	AR	51	NM_000235.4:c.398del	p.(Ser133*)	Nonsense	Homo	P
			52	NM_000235.4:c.260G > T	p.(Gly87Val)	Missense	Homo	LP
***MEFV***	Familial Mediterranean fever	AR	53	NM_000243.3:c.2076_2078del	p.(Ile692del)	Inframe del	Double-Hetero	LP
				NM_000243.3:c.2177T > C	p.(Val726Ala)	Missense		LP
***NAGLU***	Mucopolysaccharidosis type IIIB	AP	54	NM_000263.4:c.1444C > T	p.(Arg482Trp)	Missense	Homo	LP
***NPC1***	Niemann-Pick disease, type C1	AR	55	NM_000271.5:c.754del#	p.(Gln252Serfs*58)	Frameshift	Homo	P
			56	NM_000271.5:c.2045_2048delinsA#	p.(Leu682_Thr683delinsTyr)	Inframedelins	Homo	LP
			57	NM_000271.5:c.352_353del	p.(Gln119Valfs*8)	Frameshift	Homo	P
			58	NM_000271.5:c.3032_3038delinsTAGGTTTACTC#	p.(Cys1011Leufs*11)	Frameshift	Homo	LP
			59	NM_000271.5:c.2130 + 1G > C#		Splice donor	Double-Hetero	LP
				NM_000271.5:c.3530dup#	p.(ser1178Glufs*80)	Frameshift		LP
***PGM1***	Congenital disorder of glycosylation, type It	AR	60	NM_002633.3:c.1135T > G#	p.(Phen379Val)	Missense	Homo	VUS
***PHKA2***	Glycogen storage disease, type IXa1/ IXa2	XL	61	NM_000292.3:c.343A > G#	p.(Lys115Glu)	Missense	Hemi	VUS
			62	NM_000292.3:c.2807_2809del#	p.(Gly936del)	Inframe	Hemi	VUS
			63	NM_000292.3:c.2432A > G#	p.(Tyr811Cys)	Missense	Hemi	VUS
***PHKB***	Phosphorylase kinase deficiency of liver and muscle, autosomal recessive	AR	64,65	NM_000293.3:c.514-2A > C#		Splice acceptor	Homo	VUS
***PHKG2***	Glycogen storage disease IXc	AR	66	chr16:30,753,227-30757102del		CNV deletion	Homo	LP
			67	NM_000294.3:c.859C > T	p.(Gln287*)	Nonsense	Homo	LP
			68	NM_000294.3:c.377_378del#	p.(Ser126fs*)	Frameshift	Homo	LP
***PYGL***	Glycogen storage disease VI	AR	69	NM_002863.5:c.2017G > A	p.(Glu673Lys)	Missense	Homo	LP
			70	NM_002863.5:c.1768 + 1G > A		Splice donor	Homo	P
			71	NM_002863.5:c.1471C > T	p.(Arg491Cys)	Missense	Homo	LP
			72	NM_002863.5:c.361G > T#	p.(Glu121*)	Nonsense	Homo	P
			73	NM_002863.5:c.1947C > A	p.(Tyr649*)	Nonsense	Double-Hetero	P
				NM_002863.5:c.361G > T#	p.(Glu121*)	Nonsense		P
			74	NM_002863.5:c.514C > T	p.(Arg172*)	Nonsense	Homo	P
***RNF220***	Leukodystrophy, hypomyelinating, 23, with ataxia, deafness, liver dysfunction, and dilated cardiomyopathy	AR	75	NM_018150.4:c.1093C > T#	p.(Arg365Trp)	Missense	Homo	LP
***SLC17A5***	Sialic acid storage disorder, infantile	AR	76	NM_012434.5:c.1001C > G	p.(Pro334Arg)	Missense	Homo	LP
***SLC22A5***	Carnitine deficiency, systemic primary	AR	77	NM_003060.4:c.812C > G	p.(Pro271Arg)	Missense	Homo	LP
***SLC2A2***	Fanconi-Bickel syndrome	AR	78	NM_000340.2:c.1459del#	p.(Thr487Profs*27)	Frameshift	Homo	LP
			79	NM_000340.2:c.1093C > T	p.(Arg365*)	Nonsense	Homo	P
			80	NM_000340.2:c.515T > G#	p.(Val172Gly)	Missense	Homo	VUS
***SLC37A4***	Glycogen storage disease Ib/Ic	AR	81	NM_001164277.2:c.202G > A	p.(Gly68Arg)	Missense	Homo	LP
			82	NM_001164277.2:c.454G > A#	p.(Gly152Ser)	Missense	Homo	VUS
			83	NM_001164277.2:c.1309C > T	p.(Arg437*)	Nonsense	Homo	LP
			84	NM_001164277.2:c.83G > A	p.(Arg28His)	Missense	Homo	LP
***SMPD1***	Niemann-Pick disease, type A/B	AR	85	NM_000543.5:c.1624C > T	p.(Arg542*)	Nonsense	Homo	P
			86	NM_000543.5:c.955G > C	p.(Gly319Arg)	Missense	Homo	P
			87	NM_000543.5:c.1430C > T	p.(Pro477Leu)	Missense	Homo	P
***WDR19***	Nephronophthisis 13	AR	88	NM_025132.4:c.773C > T#	p.(Ser258Phe)	Missense	Double-Hetero	VUS
				NM_025132.4:c.778C > T#	p.(His260Tyr)	Missense		VUS

**Table 3:** Variants detected in Egyptian children with a primary presentation of cholestasis (*N*=83)

Gene	Disease	Inh	Patients	HGVSC	HGVSP	Variant type	Zygosity	ACMG
***ABCB11***	Cholestasis, progressive familial intrahepatic 2	AR	1,2	NM_003742.4:c.1243C > T	p.(Arg415*)	Nonsense	Homo	P
			3	NM_003742.4:c.1907A > G	p.(Glu636Gly)	Missense	Homo	LP
			4	NM_003742.4:c.2694G > A#	p.(Trp898*)	Nonsense	Homo	LP
			5	NM_003742.4:c.499G > A	p.(Ala167Thr)	Missense	Homo	LP
			6	NM_003742.4:c.1165G > C#	p.(Ala389Pro)	Missense	Homo	VUS
			7	NM_003742.4:c.1521_1527del#	p.(Phe508Profs*19)	Frameshift	Double-Hetero	LP
				NM_003742.4:c.1826_1827dup	p.(Ile610Glnfs*45)	Frameshift		P
			8	NM_003742.4:c.2087G > A#	p.(Arg696Gln)	Missense	Homo	VUS
			9	NM_003742.4:c.2629G > A	p.(Gly877Arg)	Missense	Homo	LP
			10	NM_003742.4:c.3361_3383delinsCACA#	p.(Thr1121Hisfs*11)	Frameshift	Homo	LP
			11	NM_003742.4:c.3400C > T	p.(Gln1134*)	Nonsense	Homo	P
			12	NM_003742.4:c.3802C > T	p.(Arg1268Trp)	Missense	Homo	LP
			13	NM_003742.4:c.3904G > T	p.(Glu1302*)	Missense	Homo	P
***ABCB4***	Cholestasis, progressive familial intrahepatic 3	AR	14,15	NM_000443.4:c.1493C > T#	p.(Thr498Ile)	Missense	Homo	LP
			16,17	NM_000443.4:c.1745G > A	p.(Arg582Gln)	Missense	Homo	LP
			18	NM_000443.4:c.137T > G#	p.(Phe46Cys)	Missense	Homo	LP
			19	NM_000443.4:c.3352G > T#	p.(Gln1118*)	Nonsense	Homo	LP
			20	NM_000443.4:c.1436C > T	p.(Pro479Leu)	Missense	Homo	LP
			21	NM_000443.4:c.1724T > C#	p.(Leu575Pro)	Missense	Homo	LP
			22	NM_000443.4:c.2924 + 5del#		Splice donor	Homo	VUS
			23	NM_000443.4:c.3197C > A#	p.(Ala1066Asp)	Missense	Homo	LP
			24	NM_000443.4:c.3580C > T	p.(Arg1194*)	Nonsense	Homo	LP
			25	NM_000443.4:c.430C > T	p.(Arg144*)	Nonsense	Homo	P
			26	NM_000443.4:c.628_643del#	p.(Phe210Serfs*5)	Frameshift	Homo	LP
			27	NM_000443.4:c.3197C > A#	p.(Ala1066Asp)	Missense	Double-Hetero	LP
				NM_000443.4:c.902T > C	p.(Met301Thr)	Missense		LP
***ADK***	Hypermethioninemia due to adenosine kinase deficiency	AR	28	NM_006721.4:c.312dup#	p.(Gly105Trpfs*7)	Frameshift	Homo	LP
***ATP8B1***	Cholestasis, progressive familial intrahepatic 1	AR	29	chr18:57731627_57731807dup# dup		CNV dup	Homo	VUS
			30	NM_001374385.1:c.2663C > T#	p.(Thr888Met)	Missense	Homo	VUS
			31	NM_001374385.1:c.1112G > A#	p.(Trp371*)	Nonsense	Homo	LP
			32	NM_001374385.1:c.1660G > A	p.(Asp554Asn)	Missense	Homo	P
			33	NM_001374385.1:c.1473 + 1G > C#		Splice donor	Homo	LP
***CFTR***	Cystic fibrosis	AR	34	NM_000492.4:c.1521_1523del	p.(Phe508del)	Inframe del	Homo	LP
			35	NM_000492.4:c.3909C > G	p.(Asn1303Lys)	Missense	Homo	P
			36	NM_000492.4:c.1418del	p.(Gly473Glufs*54)	Frameshift	Homo	P
			37	NM_000492.4:c.1521_1523del	p.(Phe508del)	Inframe del	Double-Hetero	LP
				NM_000492.4:c.2017G > T	p.(Gly673*)	Nonsense		P
***CLDN1***	Ichthyosis, leukocyte vacuoles and sclerosing cholangitis	AR	38	chr3:190,321,984-190388443del		CNV del	Homo	LP
***CPT1A***	CPT deficiency, hepatic, type IA	AR	39	NM_001876.4:c.879 + 5G > A#		Splice donor	Homo	VUS
***CYP21A2***	Adrenal hyperplasia, congenital, due to 21-hydroxylase deficiency	AR	40	NM_001368143.2:c.-126C > G		Intronic	Double-Hetero	P
				NM_001368143.2:c.844G > T	p.(Val282Leu)	Missense		LP
***DCDC2***	Sclerosing cholangitis, neonatal	AR	41,42	NM_016356.5:c.529dup	p.(Ile177Asnfs*20)	Frameshift	Homo	P
			43	NM_016356.5:c.290T > C#	p.(Leu97Pro)	Missense	Homo	VUS
			44	NM_016356.5:c.557 + 1G > T#		Splice donor	Homo	P
			45	NM_016356.5:c.874A > T#	p.(Lys292*)	Nonsense	Homo	LP
***DNAAF1***	Ciliary dyskinesia, primary, 13	AR	46	NM_178452.6:c.741 + 3_741 + 8del#		Splice donor	Homo	VUS
***DYNC2H1***	Short-rib thoracic dysplasia 3 with or without polydactyly	AR	47	NM_001377.3:c.10100G > A	p.(Arg3367His)	Missense	Homo	LP
***ERCC4***	Fanconi anemia, complementation group Q	AR	48	NM_005236.3:c.2651T > C#	p.(Leu884Pro)	Missense	Homo	LP
***GALT***	Galactosemia	AR	49	NM_000155.4:c.371G > A#	p.(Gly124Glu)	Missense	Homo	LP
***HNF1A***	Diabetes mellitus, insulin-dependent, 20	AD	50	NM_000545.8:c.608G > A	p.(Arg203His)	Missense	Hetero	P
***HNF4A***	Fanconi renotubular syndrome 4, with maturity-onset diabetes of the young	AD	51	NM_175914.5:c.431A > G#	p.(Tyr144Cys)	Missense	Hetero	VUS
***HSD3B7***	Bile acid synthesis defect, congenital, 1	AR	52,53,54	NM_025193.4:c.694 + 2T > G#		Splice donor	Homo	LP
			55	NM_025193.4:c.586G > A#	p.(Gly196Ser)	Missense	Homo	LP
***JAG1***	Alagille syndrome 1	AD	56	NM_000214.3:c.693_694del	p.(Arg231Serfs*10)	Frameshift	Hetero	P
			57	NM_000214.3:c.82-9C > A#		Splice acceptor	Hetero	VUS
			58	NM_000214.3:c.384G > A	p.(Trp128*)	Nonsense	Hetero	P
***KIF12***	Cholestasis, progressive familial intrahepatic, 8	AR	59	NM_138424.2:c.840G > A#	p.(Trp280*)	Nonsense	Homo	LP
			60	NM_138424.2:c.147G > A#	p.(Trp49*)	Nonsense	Homo	LP
			61	NM_138424.2:c.610G > A	p.(Val204Met)	Missense	Homo	LP
***MPV17***	Mitochondrial DNA depletion syndrome 6 (hepatocerebral type)	AR	62,63	NM_002437.5:c.284dup	p.(Phe96Leufs*17)	Frameshift	Homo	P
***MYO5B***	Cholestasis, progressive familial intrahepatic, 10	AR	64	NM_001080467.3:c.1523A > T#	p.(Asp508Val)	Missense	Homo	VUS
***NEK8***	Nephronophthisis 9	AR	65	NM_178170.3:c.35G > C#	p.(Arg12Thr)	Missense	Homo	VUS
***NOTCH2***	Alagille syndrome 2	AD	66	NM_024408.4:c.5003-1G > A#		Splice acceptor	Hetero	LP
***NPC1***	Niemann-Pick disease, type C1	AR	67	NM_000271.5:c.3637T > G	p.(Leu1213Val)	Missense	Homo	P
			68	NM_000271.5:c.1211G > A	p.(Arg404Gln)	Missense	Homo	P
***SCYL1***	Spinocerebellar ataxia, autosomal recessive 21	AR	69	NM_020680.4:c.463T > G#	p.(Trp155Gly)	Missense	Homo	VUS
***SERPINA1***	alpha-1-antitrypsin (AAT) deficiency	AR	70	NM_000295.5:c.1096G > A	p.(Glu366Lys)	Missense	Homo	LP
***SLC25A13***	Citrullinemia, type II, neonatal-onset	AR	71	NM_014251.3:c.284C > A	p.(Ala95Asp)	Missense	Homo	VUS
***SMPD1***	Niemann-Pick disease, type A/B	AR	72	NM_000543.5:c.1406A > C	p.(Tyr469Ser)	Missense	Homo	P
***SPTB***	Spherocytosis, type 2/ Elliptocytosis-3	AD	73	NM_001024858.4:c.3841C > T#	p.(Gln1281*)	Nonsense	Hetero	P
***TFAM***	Mitochondrial DNA depletion syndrome 15 (hepatocerebral type)	AR	74	NM_003201.3:c.680G > A#	p.(Arg227Gln)	Missense	Homo	VUS
***TJP2***	Cholestasis, progressive familial intrahepatic 4	AR	75	NM_004817.4:c.2229dup#	p.(Ser744Glnfs*8)	Frameshift	Homo	LP
			76	NM_004817.4:c.1978del#	p.(Glu660Argfs*6)	Frameshift	Homo	LP
			77	NM_004817.4:c.2639del	p.(Thr880Serfs*12)	Frameshift	Homo	P
			78	NM_004817.4:c.2761-6C > G#		Splice acceptor	Double-Hetero	VUS
				NM_004817.4:c.3085-2A > G#		Splice acceptor		VUS
***USP53***	Cholestasis, progressive familial intrahepatic, 7	AR	79	NM_001371395.1:c.372 + 1G > A#		Splice donor	Homo	LP
			80	NM_001371395.1:c.475_476del#	p.(Leu159Valfs*2)	Frameshift	Homo	LP
***VIPAS39***	Arthrogryposis, renal dysfunction, and cholestasis 2	AR	81	NM_001193315.2:c.1126 G > C#	p.(Ala376Pro)	Missense	Homo	VUS
***YARS1***	Infantile-onset multisystem neurologic, endocrine, and pancreatic disease 2	AR	82	NM_003680.4:c.611A > G	p.(Tyr204Cys)	Missense	Homo	VUS
***ZFYVE19***	Cholestasis, progressive familial intrahepatic, 9	AR	83	NM_001077268.2:c.513dup#	p.(Arg172Thrfs*8)	Frameshift	Homo	LP

**Table 4:** Variants detected in Egyptian children with a primary presentation of liver cell failure (*N*=8)

Gene	Disease	Inh.	Patients	HGVSC	HGVSP	Variant type	Zygosity	ACMG
***DGUOK***	Mitochondrial DNA depletion syndrome 3 (hepatocerebral type)	AR	1	NM_080916.3:c.137 A>G	p.(Asn46Ser)	Missense	Homo	LP
			2	NM_080916.3:c.128T>C	p.(Ile43Thr)	Missense	Homo	LP
***GALT***	Galactosemia	AR	3,4	NM_000155.4:c.1031 A>G#	p.(Gln344Arg)	Missense	Homo	LP
			5	NM_000155.4:c.610 C>T	p.(Arg204*)	Nonsense	Homo	P
			6	NM_000155.4:c.1009G>A#	p.(Val337Ile)	Missense	Homo	LP
***MPV17***	Mitochondrial DNA depletion syndrome 6 (hepatocerebral type)	AR	7	NM_002437.5:c.62T>G	p.(Leu21Arg)	Missense	Homo	LP
***POLG***	Mitochondrial DNA depletion syndrome 4 A (Alpers type)	AR	8	NM_002693.3:c.3100_3104+2delinsCA#		Splice donor	Double-Hetero	LP
				NM_002693.3:c.3599 C>A#	p.(Pro1200His)	Missense		LP

The pathogenicity of suspected variants was classified according to the American College of Medical Genetics and Genomics (ACMG) guidelines. All gene symbols and nomenclature were according to the HUGO Gene Nomenclature Committee (HGNC) websitehttps://www.genenames.org/. All variant nomenclature complies with the guidelines of the human genome variation society (HGVS) http://varnomen.hgvs.org/. The chosen gene transcript is the most biologically relevant transcript for each gene (MANE-Select transcript) based on the ensembl database http://www.ensembl.org/index.html

*ACMG* American college of medical genetics pathogenicity evaluation,* Inh* inheritance,* HGVSC* base change,* HGVSP* protein change

#, novel variant

**Table 5:** Variants detected in Egyptian children with a primary presentation of hyperbilirubinemic syndromes(*N*=6)

Gene	Disease	Inh.	Patients	HGVSC	HGVSP	Variant type	Zygosity	ACMG
***ABCC2***	Dubin-Johnson syndrome	AR	1	NM_000392.5:c.3258+2T>G		Splice donor	Homo	LP
			2	NM_000392.5:c.2441 C>A#	p.(Thr814Asn)	Missense	Homo	VUS
***UGT1A1***	Crigler-Najjar syndrome, type I/II	AR	3	NM_000463.3:c.1312dup#	p.(Glu438Glyfs*71)	Frameshift	Homo	LP
			4	NM_000463.3:c.824_826del	p.(Val275del)	Inframe del	Homo	LP
			5	NM_000463.3:c.1448G>A#	p.(Trp483*)	Nonsense	Homo	LP
			6	NM_000463.3:c.674T>G	p.(Val225Gly)	Missense	Double-Hetero	LP
				NM_000463.3:c.907G>A	p.(Val303Met)	Missense		VUS

The pathogenicity of suspected variants was classified according to the American College of Medical Genetics and Genomics (ACMG) guidelines. All gene symbols and nomenclature were according to the HUGO Gene Nomenclature Committee (HGNC) websitehttps://www.genenames.org/. All variant nomenclature complies with the guidelines of the human genome variation society (HGVS) http://varnomen.hgvs.org/. The chosen gene transcript is the most biologically relevant transcript for each gene (MANE-Select transcript) based on the ensembl database http://www.ensembl.org/index.html

*ACMG* American college of medical genetics pathogenicity evaluation,* Inh* inheritance,* HGVSC* base change,* HGVSP* protein change

#, novel variant;

## Discussion

The recent expanding use of next-generation sequencing technologies has aided a large number of genetically affected patients and families cut short their diagnostic odysseys, which in the past used to extend over years and up to decades in many occasions. While the prevalence of most genetic diseases is low, together, monogenic disorders account for approximately 10 in every 1000 live-births according to the World Health Organization (Fagiuoli et al. 2013). We herein report the results of a six-year-experience in the genetic diagnosis of a large cohort of Egyptian children (228 individuals) presenting with various forms of genetic liver diseases. Egypt has a peculiar Middle-Eastern and African origins, thus the genetic landscape of such disorders in Egypt is expected to be unique compared to populations in Europe, East Asia and North America. The focus in this study was the genetic landscape of monogenic liver patients in relation to their main presenting phenotypes, thus specific clinical features, other laboratory investigations or pathological findings were not presented.

A few decades ago, the majority of infants with neonatal cholestasis and a histological pattern of giant cell hepatitis were classified as “idiopathic neonatal hepatitis”. Over time, this term has been used much less frequently because modern tools allow recognition of specific genetic syndromes (Balistreri and Bezerra 2006). In the NGS era, a genetic cause of infantile cholestasis is being identified in 40–60% of patients (Nicastro and D’Antiga 2018). We detected 83% diagnostic-yield in our cholestasis cohort, which may be partially due to the higher rates of consanguinity in our cohort. In the present study, cholestatic disorders represented 44.3% of all suspected children and the diagnostic yield of tested patients was 82.2%. The most common variants in our cholestatic cohort were detected in *ABCB4*,* ABCB11* and *ATP8B1* genes causing PFIC3, PFIC2 and PFIC1, respectively. Collectively, different PFIC syndromes constituted 51.8% of patients with cholestasis and 53.8% of detected variants in this group. Pediatric cholestatic diseases often present with overlapping phenotypes and may be indistinguishable on the basis of clinical ground, biochemical markers or histological features, making accurate diagnosis challenging. Patients with PFIC3 were reported to be misdiagnosed as Wilson disease because of copper retention in some cases with cholestasis, that can result in flooding of copper in urine with penicillamine challenge test (Stalke et al. 2018; Gomez-Ospina et al. 2016; Qiu et al. 2017; Boga et al. 2015; Shneider 2011). Another value for NGS in early onset cholestasis is to identify the defect causing low-GGT PFICs (PFIC1, PFIC2, PFIC4 and the recently discovered *MYO5B*-associated cholestasis (PFIC10) (Gomez-Ospina et al. 2016; Qiu et al. 2017). Indeed, each defect bears the risk of different complications before and after transplantation (such as severe post-liver transplantation diarrhea, steatosis and graft loss in PFIC1, and the risk of hepatocellular carcinoma in the native liver or recurrent immune-mediated disease after transplantation in PFIC2 (Shneider 2009). Thus, genetic characterization is the crossroad for a decision-making about possible medical, surgical bile acid lowering treatment (e.g. biliary diversion) or towards transplantation (Nicastro and D’Antiga 2018). Moreover, the diagnosis of Alagille syndrome may not be obvious in young infants with few or incomplete syndromic features or with a biopsy without paucity of bile ducts, thus in such cases NGS will help in accurate diagnosis (Matte et al. 2010). Similar to our study, PFIC syndromes were the most prevalent diagnosis in Stalke et al. cholestasis patients; however, it was followed by bile acid synthesis defects and citrin deficiency (Stalke et al. 2018). In contrast, Nicastro and D’Antiga, reported Alagille syndrome as the predominant form followed by PFICs (Nicastro and D’Antiga 2018). Both studies were in Caucasian patients. Meanwhile, in Fang et al. which included Chinese children, the most frequent disease was citrin deficiency, which accounted for 25.4% of patients and the diagnostic rate in the cholestatic liver disease group was relatively poor at 46.0% (Fang et al. 2021). Citrin deficiency is common in East Asia and Southeast Asia and it is especially common in Northeast China (Okano et al. 2019). Since the number of genes involved in neonatal and infantile cholestasis is constantly evolving, the gene panel must be frequently updated by adding new genes. Patients with undiagnosed diseases are prime candidates for revisiting their genetic raw data, to apply the updated genetic knowledge to their target panels and their exome and genome analyses (Almes et al. 2022).

The current study included 102/228 patients presenting with organomegaly. The diagnostic yield of NGS in this group was 88/102 patients (86.3%). GSD represented the most common diagnosis (44.3%) in this group. Eighty-two disease causing variants belonging to 40 different genes were detected in children with organomegaly. The most frequent gene affecting this group was *AGL* (GSD3), followed by *PYGL* gene (GSD6), *NPC1* (NPC1) and *SLC37A4* (GSD1). The most commonly detected variant in this group was exon 28–30 deletion in the *AGL* gene. Similarly, Fang et al. reported, that the diagnostic rate of NGS in the group of patients with hepatosplenomegaly was 84.6% with GSD as the predominant etiology in Chinese pediatric patients (Fang et al. 2021). Although clinical and laboratory findings were useful in the diagnosis of GSD, genetic tests could precisely identify the exact form of GSD according to the defective gene.

Several cases with organomegaly may have overlapping clinical phenotypes. In such cases, a precise diagnosis through NGS is desirable to help improve patients’ outcome through timely and proper therapy. An example for this scenario in our cohort are four patients presenting with a GSD phenotype but two of them were diagnosed as hereditary fructose intolerance (*ALDOB* gene) and the other two had fructose 1,6 bisphosphatase deficiency (*FBP1* gene). The precise genetic diagnosis in those patients completely altered their dietary therapy.

Moreover, patients with galactosemia in our study presented with different phenotypic spectrum ranging from hepatomegaly (1 patient), infantile cholestasis (1 patient) to acute liver cell failure (4 patients). This was interesting, as those patients were not picked up by the biochemical screening for metabolic liver diseases. It is worth noting that during the 6-year-course of the study many other children with galactosemia were confirmed biochemically and were not included in this study. The same was true for the absolute majority of children with other biochemically detectable metabolic liver diseases, such as Wilson’s disease, cystic fibrosis and Niemann-Pick A/B. Furthermore, NGS overcomes the problem of securing the diagnosis in patients with high suspicion index already started on substrate-free diet, in which the blood marker rapidly disappears, and a metabolic challenge is harmful (McKiernan 2002). It could also achieve a diagnosis in subclinical/early phase of some metabolic liver diseases when clinical and biochemical hallmarks are absent or insufficient to make a diagnosis until a trigger (oxidative stress, diet or infection) produces a consistent, sometimes irreversible, metabolic derangement (Nicastro and D’Antiga 2018). In addition, some systemic diseases might secondarily produce transient cholestasis in infants such as congenital adrenal hyperplasia. NGS in this case helps to unveil the diagnosis and saves the time and extensive investigations of primary liver causes of cholestasis.

The present study included 12/228 neonates and children (5.3%) who presented with acute liver failure. The diagnostic yield of NGS in this group was 66.7%. The management of children with acute liver failure is challenging. Those children may be listed for liver transplantation before a diagnosis is determined. Reaching a timely diagnosis is of utmost importance in this scenario, because some monogenic liver diseases may respond to medical treatment, and on the other hand, others may represent a contraindication to transplantation (Hegarty et al. 2015). Furthermore, during acute decompensation, appropriate biochemical and histological evaluations can be unfeasible or ineffective because of the patient instability and the numerous confounders produced by severe organ injury. In this setting, NGS represents a pillar in the management of acute liver failure effectively identifying underlying monogenic disorders, provided the turnaround time is fast enough to impact the decision to transplant or not (Nicastro and D’Antiga 2018). This was the scenario in one of our patients with acute liver failure, who was a 5-year-old and underwent liver transplantation, and then 2 months later he developed intractable seizures and eventually died. Unfortunately, his genetic result appeared after the transplantation and came as *MPV17* pathogenic variant “Alpers syndrome”. Liver dysfunction in Alpers syndrome was reported to occur preceding seizure onset or at the terminal stage of the disease (Saneto et al. 2013). NGS may prevent futile liver transplantation in some types of mitochondrial DNA depletion syndromes or Niemann-Pick disease type C (Stalke et al. 2018). In addition, NGS helps to discriminate cases of acutely ill children in which mitochondrial DNA depletion could be secondary to acute liver injury (McKiernan et al. 2016).

It is worth noting that in the present study, genetic testing was performed only for patients whose clinical and biochemical findings failed to reach a precise diagnosis. Therefore, certain diagnoses of some relatively common monogenic liver diseases were under-represented in the current study. Examples include patients with classic clinical and radiological features of Alagille syndrome or classic biochemical findings, such as in Wilson’s disease, hepatorenal tyrosinemia or Crigler Najjar syndrome type 1. These syndromes were not commonly referred for genetic testing as their diagnosis could be reached clinically and biochemically; however, in some patients with atypical phenotypes genetic diagnosis may be needed.

Our study may have some limitations. The first is the long turnaround time to get the result of NGS up to several months, especially in the setting of a developing country, which is of essence especially in patients presenting with acute liver failure. The second limitation is the lack of proper family segregation studies, particularly for novel variants because of limited resources. Actually, in the clinical setting in Egypt, NGS is a diagnostic modality that is not available for most patients in need. Another limitation is inherent in the short-read NGS techniques used in this study, which is the inability to detect certain types of variants, such as short tandem repeats, which may cause several hepatic genetic syndromes particularly those associated with the hyperbilirubinemia phenotype (Zhou et al. 2018). This may partially explain the lower genetic diagnostic yield in this group.

Finding solutions to shorten the long turnaround time needed for the sequencing and the interpretation of large scale genomic data is mandatory for the proper and timely management of clinical emergencies. The development and free availability of accurate artificial intelligence models that can identify and prioritize variants according to their pathogenicity and phenotypic association, which is not yet fully attainable, is an important step in the right direction.

In conclusion, this study is the first to provide a bird-eye view on the landscape of pediatric monogenic liver diseases in Egypt, the Middle East and in Africa. We further enriched the global genomic knowledge with 85 novel variants invarious monogenic liver diseases. Glycogen storage disorders, particularly GSD3 and GSD6 were the most common cause of organomegaly, while progressive familial intrahepatic cholestasis, particularly PFIC3 and PFIC2 were the most common cause of cholstasis in Egyptian children. Our results can guide national health care policy makers towards the proper planning to prevent or mitigate the effects of some of the most common monogenic liver diseases in Egypt. Our genomic data here can further provide the basis for an electronic registry for monogenic liver diseases that can benefit pediatricians and pediatric hepatologists in Egypt and surrounding countries.

## Supplementary Information

Below is the link to the electronic supplementary material.

Supplementary Material 1

## Data Availability

Relevant data are provided in text and supplementary materials and further clinical or genetic data are available from the corresponding author upon reasonable request.
