# Supplementary material for: Unfolding the genetic map of monogenic liver diseases in Egypt
Source: Hum Genet. 2025 Oct 30;144(11-12):1053–70. doi: 10.1007/s00439-025-02776-4 (PMC12689692; doi:10.1007/s00439-025-02776-4)
Supplement: Supplementary file 1 — Supplementary Material 1 [file 439_2025_2776_MOESM1_ESM.docx]

**Supplementary materials**

**Title: Unfolding the Genetic Map of Monogenic Liver Diseases in Egypt**

**Hanaa El-Karaksy^1^, Engy A. Mogahed^1^, Sherif Baroudy^1^, Haytham Ghita^1^, Afaf Enayet^1^, Marwa El-Sharkawy^2^, Noha A. Radwan^2^, Heba Hosny^3^, Mohamed A. Elmonem^2^**

^1^ Department of Pediatrics, Faculty of Medicine, Cairo University, Cairo, Egypt

^2^ Department of Clinical and Chemical Pathology, Faculty of Medicine, Cairo University, Cairo, Egypt

^3^ Fellow of Medical and Clinical Genetics, National Institute of Neuro-Motor System, Egypt

**Corresponding author:**

***Corresponding author:** Dr. Mohamed A. Elmonem

**Address:** Cairo University Children's Hospital, New Clinics building, Cairo 11628, Egypt.

**Phone:** +201146870824 **Email:** [mohamed.abdelmonem@kasralainy.edu.eg](mailto:mohamed.abdelmonem@kasralainy.edu.eg)

**ORCID:** [0000-0002-3154-1948](https://orcid.org/0000-0002-3154-1948)

**Supplementary Table 1.** A summary of the numbers of diagnosed children and the number of variants detected in each phenotype.

| **Clinical phenotypes** | **Patients suspected** | **Patients confirmed** | **Genes Affected** | **Variants detected** | **Novel variants** | **Reported variants** |
| --- | --- | --- | --- | --- | --- | --- |
|  |  |  |  |  |  |  |
| Organomegaly | 102 | 88 | 40 | 82 | 33 | 49 |
| Cholestasis | 101 | 83 | 34 | 78 | 45 | 33 |
| Liver cell failure | 12 | 8 | 4 | 8 | 4 | 4 |
| Hyperbilirubinemia | 13 | 6 | 2 | 7 | 3 | 4 |
| **Total** | **228** | **185** | **72** | **175** | **85** | **90** |

**Supplementary Table 2.** Pathogenicity scoring and ACMG justification for all detected variants in Egyptian children with monogenic liver diseases

| **Gene** | **Transcript**  **(MANE select)** | **Chromosomal location (GRCh38)** | **HGVSC** | **HGVSP** | **Variant impact** | **ACMG** | **ACMG justification** | **gnomAD**  **MAF** | **REVEL** | **CADD** | **SpliceAI** | **Novel/ Reported** |
| --- | --- | --- | --- | --- | --- | --- | --- | --- | --- | --- | --- | --- |
|  |  |  |  |  |  |  |  |  |  |  |  |  |
| ***ABCA1*** | NM_005502.4 | chr9-104796118 C>T | c.5317G>A | p.(Val1773Met) | Missense | R | PM2, PP3 | 0.00001 | 0.678 | 25.6 |  | Novel |
| ***ABCB11*** | NM_003742.4 | chr2-168976642 G>A | c.1243C>T | p.(Arg415*) | Nonsense | P | PVS1, PM2, PM3 | 0.000001 |  |  |  | Reported |
|  |  | chr2-168969454 T>C | c.1907A>G | p.(Glu636Gly) | Missense | LP | PM2, PM3, PM5, PP3 | 0.0000006 | 0.936 | 27.4 |  | Reported |
|  |  | chr2-168936350 C>T | c.2694G>A | p.(Trp898*) | Nonsense | LP | PVS1, PM2 | 0.0000006 |  |  |  | Novel |
|  |  | chr2-168995461 C>T | c.499G>A | p.(Ala167Thr) | Missense | LP | PM2, PM3, PP3 | 0.00002 | 0.796 | 24 |  | Reported |
|  |  | chr2-168979898 C>G | c.1165G>C | p.(Ala389Pro) | Missense | R | PM2, PP3 | 0.00 | 0.930 | 29.2 |  | Novel |
|  |  | chr2-168971957 TAGAGAAC>T | c.1521_1527del | p.(Phe508Profs*19) | Frameshift | LP | PVS1, PM2 | 0.00 |  |  |  | Novel |
|  |  | chr2-168969533 T>TTG | c.1826_1827dup | p.(Ile610Glnfs*45) | Frameshift | P | PVS1, PM2, PM3 | 0.00 |  |  |  | Reported |
|  |  | chr2-168964297 C>T | c.2087G>A | p.(Arg696Gln) | Missense | R | PM2, PM5 | 0.00001 | 0.356 | 24.7 |  | Novel |
|  |  | chr2-168936415 C>T | c.2629G>A | p.(Gly877Arg) | Missense | LP | PM2, PP3, PP5 | 0.000005 | 0.941 | 31 |  | Reported |
|  |  | chr2-168930693_168930715delins | c.3361_3383delinsCACA | p.(Thr1121Hisfs*11) | Frameshift | LP | PVS1, PM2 | 0.00 |  |  |  | Novel |
|  |  | chr2-168930676 G>A | c.3400C>T | p.(Gln1134*) | Nonsense | P | PVS1, PM2, PM3 | 0.00 |  |  |  | Reported |
|  |  | chr2-168923786 G>A | c.3802C>T | p.(Arg1268Trp) | Missense | LP | PM2, PM3, PM5, PP3 | 0.000004 | 0.882 | 33 |  | Reported |
|  |  | chr2-168923684 C>A | c.3904G>T | p.(Glu1302*) | Nonsense | P | PVS1, PM2, PM3 | 0.00 |  |  |  | Reported |
| ***ABCB4*** | NM_000443.4 | chr7-87440266 G>A | c.1493C>T | p.(Thr498Ile) | Missense | LP | PM1, PM2, PP2, PP3 | 0.00 | 0.789 | 26.7 |  | Novel |
|  |  | chr7-87431552 C>T | c.1745G>A | p.(Arg582Gln) | Missense | LP | PM1, PM2, PM5, PP3, PP5 | 0.000004 | 0.787 | 28.5 |  | Reported |
|  |  | chr7-87462907 A>C | c.137T>G | p.(Phe46Cys) | Missense | LP | PM2, PP2, PP3 | 0.00 | 0.938 | 28.9 |  | Novel |
|  |  | chr7-87406422 C>A | c.3352G>T | p.(Gln1118*) | Nonsense | LP | PVS1, PM2 | 0.00 |  |  |  | Novel |
|  |  | chr7-87440323 G>A | c.1436C>T | p.(Pro479Leu) | Missense | LP | PS4, PM1, PM2, PP3 | 0.000003 | 0.896 | 32 |  | Reported |
|  |  | chr7-87439674 A>G | c.1724T>C | p.(Leu575Pro) | Missense | LP | PM2, PP2, PP3 | 0.00 | 0.964 | 33 |  | Novel |
|  |  | chr7-87411887 AC>A | c.2924+5del |  | Splicing | R | PM2, PP3 | 0.00 |  |  | 0.59 | Novel |
|  |  | chr7-87408119 G>T | c.3197C>A | p.(Ala1066Asp) | Missense | LP | PM2, PP2, PP3 | 0.00 | 0.980 | 32 |  | Novel |
|  |  | chr7-87403188 G>A | c.3580C>T | p.(Arg1194*) | Nonsense | LP | PVS1, PM2 | 0.000003 |  |  |  | Reported |
|  |  | chr7-87453050 G>A | c.430C>T | p.(Arg144*) | Nonsense | P | PVS1, PS4, PM2 | 0.000004 |  |  |  | Reported |
|  |  | chr7-87451687_87451702del | c.628_643del | p.(Phe210Serfs*5) | Frameshift | LP | PVS1, PM2 | 0.00 |  |  |  | Novel |
|  |  | chr7-87447137 A>G | c.902T>C | p.(Met301Thr) | Missense | LP | PM2, PP2, PP3, PP5 | 0.00 | 0.678 | 24.9 |  | Reported |
| ***ABCC2*** | NM_000392.5 | chr10-99832133 T>G | c.3258+2T>G |  | Splicing | LP | PVS1, PM2, PP5 | 0.0000006 |  |  | 0.99 | Reported |
|  |  | chr10-99819090 C>A | c.2441C>A | p.(Thr814Asn) | Missense | R | PM2, PP3 | 0.0000006 | 0.811 | 29.3 |  | Novel |
| ***ABCC6*** | NM_001171.6 | chr16-16157722 G>A | c.3823C>T | p.(Arg1275*) | Nonsense | P | PVS1, PS4, PM2 | 0.00002 |  |  |  | Reported |
| ***ADK*** | NM_006721.4 | chr10-74394173 A>AT | c.312dup | p.(Gly105Trpfs*7) | Frameshift | LP | PVS1, PM2 | 0.00 |  |  |  | Novel |
| ***AGL*** | NM_000642.3 | chr1-99910707_99913753del 1-99884612 C>T | Exon 28-30 deletion |  | CNV del | P | PVS1, PM2, PM5 | 0.00 |  |  |  | Reported |
|  |  | chr1-99884612 C>T | c.2590C>T | p.(Arg864*) | Nonsense | P | PVS1, PS3, PM2, PM3 | 0.00001 |  |  |  | Reported |
|  |  | chr1-99877806 C>G | c.1589C>G | p.(Ser530*) | Nonsense | P | PVS1, PM2, PM3 | 0.00 |  |  |  | Reported |
|  |  | chr1-99880755 T>C | c.1859T>C | p.(Leu620Pro) | Missense | P | PM2, PM3, PM5, PP3 | 0.00 | 0.95 | 26.2 |  | Reported |
|  |  | chr1-99884393 G>T | c.2488G>T | p.(Glu830*) | Nonsense | LP | PVS1, PM2 | 0.00 |  |  |  | Novel |
|  |  | chr1-99913557 G>A | c.3980G>A | p.(Trp1327*) | Nonsense | P | PVS1, PM2, PM3 | 0.00009 |  |  |  | Reported |
|  |  | chr1-99910824 CAG>C | c.3816_3817del | p.(Gly1273Asnfs*18) | Frameshift | P | PVS1, PM2, PM3 | 0.000005 |  |  |  | Reported |
|  |  | chr1-99891705 ACT>A | c.3052_3053del | p.(Leu1018Serfs*51) | Frameshift | LP | PVS1, PM2 | 0.00 |  |  |  | Novel |
|  |  | chr1-99861676 C>T | c.256C>T | p.(Gln86*) | Nonsense | LP | PVS1, PM2 | 0.00003 |  |  |  | Reported |
|  |  | chr1-99881561_99881588dup | c.2179_2218dup | p.(His740Argfs*12) | Frameshift | LP | PVS1, PM2 | 0.00 |  |  |  | Novel |
| ***ALDOB*** | NM_000035.4 | chr9-101425452 C>T | c.799+1G>A |  | Splicing | P | PVS1, PM2, PM3 | 0.00 |  |  | 1.0 | Reported |
|  |  | chr9-101430878 G>A | c.10C>T | p.(Arg4*) | Nonsense | P | PVS1, PM2, PM3, PP1 | 0.00001 |  |  |  | Reported |
| ***ATP7B*** | NM_000053.4 | chr13-51937395 T>C | c.3904-2A>G |  | Splicing | P | PVS1, PM2, PM3 | 0.00 |  |  | 0.99 | Reported |
|  |  | chr13-51973930 C>A | c.1285+5G>T |  | Splicing | P | PM2, PM3, PP3 | 0.00008 |  |  | 0.78 | Reported |
|  |  | chr13-51942523 G>A | c.3275C>T | p.(Thr1092Met) | Missense | LP | PM1, PM2, PM5, PP2 | 0.00006 | 0.594 | 17.8 |  | Reported |
| ***ATP8B1*** | NM_001374385.1 | chr18:57731627_57731807dup | Exon-2 duplication |  | CNV dup | R | PM2 | 0.00 |  |  |  | Novel |
|  |  | chr18-57661218 G>A | c.2663C>T | p.(Thr888Met) | Missense | R | PM2, PP3 | 0.000003 | 0.911 | 33 |  | Novel |
|  |  | chr18-57691915 C>T | c.1112G>A | p.(Trp371*) | Nonsense | LP | PVS1, PM2 | 0.0000006 |  |  |  | Novel |
|  |  | chr18-57674993 C>T | c.1660G>A | p.(Asp554Asn) | Missense | P | PS3, PS4, PM2, PP1, PP3 | 0.000006 | 0.683 | 29.7 |  | Reported |
|  |  | chr18-57685071 C>G | c.1473+1G>C |  | Splicing | LP | PVS1, PM2 | 0.00 |  |  | 1.0 | Novel |
| ***CFTR*** | NM_000492.4 | chr7-117559590 ATCT>A | c.1521_1523del | p.(Phe508del) | Inframe del | LP | PS1, PM2, PM4, PM5, PP5 | 0.01 | 0.731 | 22 |  | Reported |
|  |  | chr7-117652877 C>G | c.3909C>G | p.(Asn1303Lys) | Missense | P | PS1, PS3, PM1, PM2, PM3, PP3 | 0.0001 | 0.830 | 22.2 |  | Reported |
|  |  | chr7-117559486 TG>T | c.1418del | p.(Gly473Glufs*54) | Frameshift | P | PVS1, PM2, PM3 | 0.000006 |  |  |  | Reported |
|  |  | chr7-117592184 G>T | c.2017G>T | p.(Gly673*) | Nonsense | P | PVS1, PS4, PM2 | 0.0000006 |  |  |  | Reported |
| ***CLDN1*** | NM_021101.5 | chr3:190321984-190388443del |  |  | CNV del | LP | PVS1, PM2 | 0.00 |  |  |  | Reported |
|  |  | chr3-190322032 T>C | c.175A>G | p.(Thr59Ala) | Missense | R | PM2, PP3 | 0.00 | 0.864 | 28.8 |  | Novel |
| ***COG4*** | NM_015386.3 | chr16-70483882 C>T | c.1798G>A | p.(Val600Met) | Missense | R | PM2 | 0.00004 | 0.411 | 23.0 |  | Novel |
| ***CPT1A*** | NM_001876.4 | chr11-68794799 C>T | c.879+5G>A |  | Splicing | R | PM2, PP3 |  |  |  | 0.93 | Novel |
| ***CYP21A2*** | NM_001368143.2 | chr6-32039081 C>G | c.-126C>G |  | Splicing | P | PS3, PM2, PM3 | 0.0024 |  |  | 0.76 | Reported |
|  |  | chr6-32040110 G>T | c.844G>T | p.(Val282Leu) | Missense | LP | PM1, PM2, PP1, PP5 | 0.005 | 0.331 | 23.4 |  | Reported |
| ***DCDC2*** | NM_016356.5 | chr6-24301742 A>AT | c.529dup | p.(Ile177Asnfs*20) | Frameshift | P | PVS1, PM2, PM3 | 0.00001 |  |  |  | Reported |
|  |  | chr6-24357461 A>G | c.290T>C | p.(Leu97Pro) | Missense | R | PM2, PP3 | 0.00 | 0.851 | 26.4 |  | Novel |
|  |  | chr6-24301714 C>A | c.557+1G>T |  | Splicing | P | PVS1, PM2, PM3 | 0.00 |  |  | 0.88 | Novel |
|  |  | chr6-24278097 T>A | c.874A>T | p.(Lys292*) | Nonsense | LP | PVS1, PM2 | 0.0000006 |  |  |  | Novel |
| ***DDX41*** | NM_016222.4 | chr5-177512511 C>A | c.1534G>T | p.(Glu512*) | Nonsense | LP | PVS1, PM2 | 0.00 |  |  |  | Novel |
| ***DGUOK*** | NM_080916.3 | chr2-73927047 A>G | c.137A>G | p.(Asn46Ser) | Missense | LP | PM2, PM3, PP2, PP3 | 0.00001 | 0.800 | 26.6 |  | Reported |
|  |  | chr2-73927038 T>C | c.128T>C | p.(Ile43Thr) | Missense | LP | PM2, PP2, PP3 | 0.00 | 0.892 | 29.1 |  | Reported |
| ***DLD*** | NM_000108.5 | chr7-107915506 G>T | c.685G>T | p.(Gly229Cys) | Missense | P | PM2, PM3, PP2, PP3 | 0.00014 | 0.681 | 34 |  | Reported |
| ***DNAAF1*** | NM_178452.6 | chr16-84155751 TAAAAAA>T | c.741+3_741+8del |  | Splicing | R | PM2, PP3 | 0.00 |  |  | 0.92 | Novel |
| ***DYNC2H1*** | NM_001377.3 | chr11-103253342 G>A | c.10100G>A | p.(Arg3367His) | Missense | LP | PM1, PM2, PM3, PM5 | 0.00003 | 0.565 | 30 |  | Reported |
| ***ERCC4*** | NM_005236.3 | chr16-13948247 T>C | c.2651T>C | p.(Leu884Pro) | Missense | R | PM2, PP3 | 0.00 | 0.760 | 25.5 |  | Novel |
| ***ETFDH*** | NM_004453.4 | chr4-158695619 A>C | c.807A>C | p.(Gln269His) | Missense | P | PM1, PM2, PM3, PM5, PP2, PP3 | 0.00 | 0.834 | 18.9 |  | Reported |
| ***FAH*** | NM_000137.4 | chr15-80173016 C>T | c.709C>T | p.(Arg237*) | Nonsense | P | PVS1, PM2, PM3 | 0.000018 |  |  |  | Reported |
|  |  | chr15-80168263 G>T | c.554-1G>T |  | Splicing | P | PVS1, PS3, PM2, PM3 | 0.00019 |  |  | 0.99 | Reported |
|  |  | chr15-80168093 T>G | c.497T>G | p.(Val166Gly) | Missense | LP | PS3, PM2, PM3, PP3 | 0.000002 | 0.751 | 23.9 |  | Reported |
|  |  | chr15-80158171 G>A | c.192+1G>A |  | Splicing | LP | PVS1, PM2, PM5 | 0.0000006 |  |  | 0.99 | Novel |
| ***FARSA*** | NM_004461.3 | chr19-12924512 G>A | c.1210C>T | p.(Arg404Cys) | Missense | R | PM2, PP3 | 0.00006 | 0.904 | 34 |  | Reported |
|  |  | chr19-12924242 C>T | c.1297G>A | p.(Gly433Arg) | Missense | R | PM2, PP3 | 0.00002 | 0.705 | 25.6 |  | Novel |
| ***FBP1*** | NM_000507.4 | chr9:93639344-94639132del |  |  | CNV del | LP | PVS1, PM2 | 0.00 |  |  |  | Reported |
|  |  | chr9-94617808 C>A | c.386G>T | p.(Cys129Phe) | Missense | LP | PM1, PM2, PP3 | 0.00 | 0.773 | 24.5 |  | Novel |
| ***G6PC*** | NM_000151.4 | chr17-42903947 C>T | c.247C>T | p.(Arg83Cys) | Missense | LP | PM1, PM2, PM3, PM5, PP3 | 0.0004 | 0.829 | 29.3 |  | Reported |
| ***GALT*** | NM_000155.4 | chr9-34647858 C>T | c.404C>T | p.(Ser135Leu) | Missense | LP | PS3, PM1, PM2, PM3, PP5, PP3 | 0.00017 | 0.921 | 30 |  | Reported |
|  |  | chr9-34647699 G>A | c.371G>A | p.(Gly124Glu) | Missense | LP | PM1, PM2, PP2, PP3 | 0.00 | 0.968 | 32 |  | Novel |
|  |  | chr9-34649536 A>G | c.1031A>G | p.(Gln344Arg) | Missense | LP | PM1, PM2, PM5, PP2, PP3 | 0.0000006 | 0.948 | 26.8 |  | Novel |
|  |  | chr9-34648379 C>T | c.610C>T | p.(Arg204*) | Nonsense | P | PVS1, PS3, PM2, PM3 | 0.00002 |  |  |  | Reported |
|  |  | chr9-34649514 G>A | c.1009G>A | p.(Val337Ile) | Missense | LP | PM1, PM2, PP2, PP3 | 0.00 | 0.929 | 28 |  | Novel |
| ***GBA*** | NM_000157.4 | chr1-155235252 A>G | c.1448T>C | p.(Leu483Pro) | Missense | P | PS3, PM1, PM2, PM3, PM5, PP3 | 0.00009 | 0.858 | 25.3 |  | Reported |
|  |  | chr1-155235828 A>C | c.1241T>G | p.(Val414Gly) | Missense | LP | PM1, PM2, PM5, PP3 | 0.0000006 | 0.942 | 24.7 |  | Novel |
| ***GBE1*** | NM_000158.4 | chr3-81649875 G>A | c.476C>T | p.(Pro159Leu) | Missense | R | PM2, PP3 | 0.000007 | 0.722 | 33 |  | Novel |
| ***GLB1*** | NM_000404.4 | chr3-33068258 CTCTT>C | c.569_572del | p.(Lys190Serfs*27) | Frameshift | P | PVS1, PM2, PP3 | 0.0000006 |  |  |  | Reported |
| ***GPD1*** | NM_005276.4 | chr12-50107760 G>A | c.806G>A | p.(Arg269Gln) | Missense | LP | PM2, PM5, PP3, PP5 | 0.00005 | 0.783 | 33 |  | Reported |
| ***HBB*** | NM_000518.5 | chr11-5226924 A>G | c.92+6T>C |  | Splicing | P | PS3, PM2, PM3, PP3 | 0.00007 |  |  | 0.63 | Reported |
| ***HMGCS2*** | NM_005518.4 | chr1-119755496 T>A | c.1118A>T | p.(Tyr373Phe) | Missense | R | PM2, PP2 | 0.000002 | 0.196 | 17.3 |  | Novel |
| ***HNF1A*** | NM_000545.8: | chr12-120993601 G>A | c.608G>A | p.(Arg203His) | Missense | P | PS3, PS4, PM1, PM2, PM5, PP3 | 0.000001 | 0.958 | 34 |  | Reported |
| ***HNF4A*** | NM_175914.5 | chr20-44413739 A>G | c.431A>G | p.(Tyr144Cys) | Missense | R | PM2, PP2, PP3 | 0.000002 | 0.773 | 27.4 |  | Novel |
| ***HSD3B7*** | NM_025193.4 | chr16-30987004 T>G | c.694+2T>G |  | Splicing | LP | PVS1, PM2 | 0.00 |  |  | 0.98 | Novel |
|  |  | chr16-30986894 G>A | c.586G>A | p.(Gly196Ser) | Missense | LP | PM2, PP2, PP3 | 0.000004 | 0.968 | 35 |  | Novel |
| ***IL18BP*** | NM_001039660.2 | chr11-71999995 GAC>G | c.15_16del | p.(His5Glnfs*40) | Frameshift | R | PVS1, PM2 | 0.00 |  |  |  | Novel |
| ***JAG1*** | NM_000214.3 | chr20-10658467 CCT>C | c.693_694del | p.(Arg231Serfs*10) | Frameshift | P | PVS1, PS4, PM2 | 0.00 |  |  |  | Reported |
|  |  | chr20-10673015 G>T | c.82-9C>A |  | Splicing | R | PM2, PP3 | 0.00 |  |  | 0.95 | Novel |
|  |  | chr20-10672704 C>T | c.384G>A | p.(Trp128*) | Nonsense | P | PVS1, PM2, PP5 | 0.00 |  |  |  | Reported |
| ***KIF12*** | NM_138424.2 | chr9-114094240 C>T | c.840G>A | p.(Trp280*) | Nonsense | LP | PVS1, PM2 | 0.00 |  |  |  | Novel |
|  |  | chr9-114097386 C>T | c.147G>A | p.(Trp49*) | Nonsense | LP | PVS1, PM2 | 0.0000006 |  |  |  | Novel |
|  |  | chr9-114095118 C>T | c.610G>A | p.(Val204Met) | Missense | LP | PS4, PM2 | 0.000015 | 0.632 | 31 |  | Reported |
| ***LIPA*** | NM_000235.4 | chr10-89228229 TG>T | c.398del | p.(Ser133*) | Nonsense | P | PVS1, PM2, PP5 | 0.00004 |  |  |  | Reported |
|  |  | chr10-89228368 C>A | c.260G>T | p.(Gly87Val) | Missense | LP | PS3, PM1, PM2, PM3, PP3 | 0.000003 | 0.899 | 28.5 |  | Reported |
| ***MEFV*** | NM_000243.3 | chr16-3243408 CATT>C | c.2076_2078del | p.(Ile692del) | Inframe del | LP | PS4, PM1, PM2, PM4 | 0.000001 |  |  |  | Reported |
|  |  | chr16-3243310 A>G | c.2177T>C | p.(Val726Ala) | Missense | LP | PM1, PM2, PP5 | 0.0013 | 0.350 |  |  | Reported |
| ***MPV17*** | NM_002437.5 | chr2-27312584 G>GC | c.284dup | p.(Phe96Leufs*17) | Frameshift | P | PVS1, PM2, PM3 | 0.00001 |  |  |  | Reported |
|  |  | chr2-27322456 A>C | c.62T>G | p.(Leu21Arg) | Missense | LP | PM2, PP2, PP3 | 0.00 | 0.919 | 30 |  | Reported |
| ***MYO5B*** | NM_001080467.3 | chr18-49962288 T>A | c.1523A>T | p.(Asp508Val) | Missense | R | PM2, PP3 | 0.00 | 0.916 | 26.7 |  | Novel |
| ***NAGLU*** | NM_000263.4 | chr17-42543450 C>T | c.1444C>T | p.(Arg482Trp) | Missense | LP | PS4, PM1, PM2, PM5, PP3 | 0.000006 | 0.900 | 23.7 |  | Reported |
| ***NEK8*** | NM_178170.3 | chr17-28728848 G>C | c.35G>C | p.(Arg12Thr) | Missense | R | PM2, PP3 | 0.00 | 0.560 | 33 |  | Novel |
| ***NOTCH2*** | NM_024408.4 | chr1-119922447 C>T | c.5003-1G>A |  | Splicing | LP | PVS1, PM2 | 0.00 |  |  | 0.98 | Novel |
| ***NPC1*** | NM_000271.5 | chr18-23533472 A>C | c.3637T>G | p.(Leu1213Val) | Missense | P | PS3, PM1, PM2, PM3, PM5, PP3 | 0.000007 | 0.857 | 22.1 |  | Reported |
|  |  | chr18-23556358 C>T | c.1211G>A | p.(Arg404Gln) | Missense | P | PS3, PM1, PM2, PM3, PM5, PP3 | 0.00002 | 0.897 | 34 |  | Reported |
|  |  | chr18-23560357 TG>T | c.754del | p.(Gln252Serfs*58) | Frameshift | P | PVS1, PM2, PM3 | 0.00 |  |  |  | Novel |
|  |  | chr18-23544426 GTCA>T | c.2045_2048delinsA | p.(Leu682_Thr683delinsTyr) | Inframe | LP | PM1, PM2, PM4 | 0.00 | 0.933 | 28.7 |  | Novel |
|  |  | chr18-23568932 ACT>A | c.352_353del | p.(Gln119Valfs*8) | Frameshift | P | PVS1, PM2, PM3 | 0.000004 |  |  |  | Reported |
|  |  | chr18-23538545 TTGCCAC>GAGTAAACCTA | c.3032_3038delinsTAGGTTTACTC | p.(Cys1011Leufs*11) | Frameshift | LP | PVS1, PM2 | 0.00 |  |  |  | Novel |
|  |  | chr18-23544343 C>G | c.2130+1G>C |  | Splicing | LP | PVS1, PM2 | 0.00 |  |  | 1.0 | Novel |
|  |  | chr18-23534506 C>CA | c.3530dup | p.(ser1178Glufs*80) | Frameshift | LP | PVS1, PM2 | 0.00 |  |  |  | Novel |
| ***PGM1*** | NM_002633.3 | chr1-63638737 T>G | c.1135T>G | p.(Phen379Val) | Missense | R | PM2, PP3 | 0.00 | 0.784 | 31 |  | Novel |
| ***PHKA2*** | NM_002633.3 | chrX-18951215 T>C | c.343A>G | p.(Lys115Glu) | Missense | R | PM2, PP3 | 0.00 | 0.918 | 25.8 |  | Novel |
|  |  | chrX-18905856 TCTC>T | c.2807_2809del | p.(Gly936del) | Inframe del | R | PM2, PM4 | 0.000005 | 0.866 | 25.9 |  | Novel |
|  |  | chrX-18907985 T>C | c.2432A>G | p.(Tyr811Cys) | Missense | R | PM2, PP3 | 0.00002 | 0.824 | 24.1 |  | Novel |
| ***PHKB*** | NM_000293.3 | chr16-47515519 A>C | c.514-2A>C |  | Splicing | LP | PVS1, PM2 | 0.00 |  |  | 1.0 | Novel |
| ***PHKG2*** | NM_000294.3 | chr16:30753227-30757102del | Exon 5-10 deletion |  | CNV del | LP | PVS1, PM2 | 0.00 |  |  |  | Novel |
|  |  | chr16-30756647 C>T | c.859C>T | p.(Gln287*) | Nonsense | LP | PVS1, PM2 | 0.000002 |  |  |  | Reported |
|  |  | chr16-30753277 CCT>C | c.377_378del | p.(Ser126fs*) | Nonsense | LP | PVS1, PM2 | 0.00 |  |  |  | Novel |
| ***POLG*** | NM_002693.3 | chr15-89319226 ACTTCCT>TG | c.3100_3104+2delinsCA |  | Splicing | LP | PVS1, PM2 | 0.00 |  |  | 0.99 | Novel |
|  |  | chr15-89317420 G>T | c.3599C>A | p.(Pro1200His) | Missense | LP | PM2, PP2, PP3 | 0.00 | 0.919 | 28.2 |  | Novel |
| ***PYGL*** | NM_002863.5 | chr14-50910055 C>T | c.2017G>A | p.(Glu673Lys) | Missense | LP | PM2, PP3 | 0.00003 | 0.978 | 32 |  | Reported |
|  |  | chr14-50912155 C>T | c.1768+1G>A |  | Splicing | P | PVS1, PM2, PM3 | 0.000017 |  |  | 1.0 | Reported |
|  |  | chr14-50914748 G>A | c.1471C>T | p.(Arg491Cys) | Missense | LP | PM2, PP3, PP5 | 0.000017 | 0.948 | 34 |  | Reported |
|  |  | chr14-50935170 C>A | c.361G>T | p.(Glu121*) | Nonsense | P | PVS1, PM2, PP5 | 0.00 |  |  |  | Novel |
|  |  | chr14-50911752 G>T | c.1947C>A | p.(Tyr649*) | Nonsense | P | PVS1, PM2, PP5 | 0.000012 |  |  |  | Reported |
|  |  | chr14-50931687 G>A | c.514C>T | p.(Arg172*) | Nonsense | P | PVS1, PM2, PM3 | 0.000003 |  |  |  | Reported |
| ***RNF220*** | NM_018150.4 | chr1-44636129 C>T | c.1093C>T | p.(Arg365Trp) | Missense | LP | PM2, PM5, PP3 | 0.000002 | 0.843 | 26.1 |  | Novel |
| ***SCYL1*** | NM_020680.4 | chr11-65526211 T>G | c.463T>G | p.(Trp155Gly) | Missense | R | PM2, PP3 | 0.00 | 0.795 | 33 |  | Novel |
| ***SERPINA1*** | NM_000295.5 | chr14-94378610 C>T | c.1096G>A | p.(Glu366Lys) | Missense | LP | PM5, PP3, PP5 | 0.0015 | 0.686 | 25.7 |  | Reported |
| ***SLC17A5*** | NM_012434.5 | chr6-73615425 G>C | c.1001C>G | p.(Pro334Arg) | Missense | LP | PS3, PM2, PM3, PP3 | 0.00 | 0.779 | 29.2 |  | Reported |
| ***SLC22A5*** | NM_003060.4 | chr5-132385415 C>G | c.812C>G | p.(Pro271Arg) | Missense | LP | PS3, PM1, PM2, PM3, PP3 | 0.00 | 0.778 | 31 |  | Reported |
| ***SLC25A13*** | NM_014251.3 | chr7-96234846 G>T | c.284C>A | p.(Ala95Asp) | Missense | R | PM2, PP3 | 0.000005 | 0.807 | 27.4 |  | Reported |
| ***SLC2A2*** | NM_000340.2 | chr3-170998018 GT>G | c.1459del | p.(Thr487Profs*27) | Frameshift | LP | PVS1, PM2 | 0.00 |  |  |  | Novel |
|  |  | chr3-170999142 G>A | c.1093C>T | p.(Arg365*) | Nonsense | P | PVS1, PS4, PM2 | 0.000014 |  |  |  | Reported |
|  |  | chr3-171007245 A>C | c.515T>G# | p.(Val172Gly) | Missense | R | PM2, PP3 | 0.00 | 0.765 | 24.7 |  | Novel |
| ***SLC37A4*** | NM_001164277.2 | chr11-119028373 C>T | c.202G>A | p.(Gly68Arg) | Missense | LP | PS3, PM1, PM2, PM3 | 0.000002 |  | 32 |  | Reported |
|  |  | chr11-119027800 C>T | c.454G>A# | p.(Gly152Ser) | Missense | R | PM2, PP2 | 0.00 |  | 22.8 |  | Novel |
|  |  | chr11-119024957 G>A | c.1309C>T | p.(Arg437*) | Nonsense | LP | PVS1, PM2, PM3 | 0.00002 |  |  |  | Reported |
|  |  | chr11-119029287 C>T | c.83G>A | p.(Arg28His) | Missense | LP | PM1, PM2, PM3, PM5 | 0.000009 |  | 32 |  | Reported |
| ***SMPD1*** | NM_000543.5 | chr11-6394335 C>T | c.1624C>T | p.(Arg542*) | Nonsense | P | PVS1, PM2, PM3 | 0.00002 |  |  |  | Reported |
|  |  | chr11-6392020 G>C | c.955G>C | p.(Gly319Arg) | Missense | P | PM1, PM2, PM3, PM5, PP3 | 0.000002 | 0.632 | 33 |  | Reported |
|  |  | chr11-6393985 C>T | c.1430C>T | p.(Pro477Leu) | Missense | P | PM1, PM2, PM3, PM5, PP3 | 0.00002 | 0.840 | 29.3 |  | Reported |
|  |  | chr11-6393961 A>C | c.1406A>C | p.(Tyr469Ser) | Missense | P | PS3, PM1, PM2, PM3, PM5, PP3 | 0.00 | 0.947 | 28 |  | Reported |
| ***SPTB*** | NM_001024858.4 | chr14-64785551 G>A | c.3841C>T | p.(Gln1281*) | Nonsense | P | PVS1, PS4, PM2 | 0.000001 |  |  |  | Novel |
| ***TFAM*** | NM_003201.3 | chr10-58395013 G>A | c.680G>A | p.(Arg227Gln) | Missense | R | PM2 | 0.00 | 0.554 | 23.3 |  | Novel |
| ***TJP2*** | NM_004817.4 | chr9-69237092 T>TC | c.2229dup | p.(Ser744Glnfs*8) | Frameshift | LP | PVS1, PM2 | 0.00 |  |  |  | Novel |
|  |  | chr9-69236128 AG>A | c.1978del | p.(Glu660Argfs*6) | Frameshift | LP | PVS1, PM2 | 0.00 |  |  |  | Novel |
|  |  | chr9-69240126 AC>A | c.2639del | p.(Thr880Serfs*12) | Frameshift | P | PVS1, PS4, PM2 | 0.000004 |  |  |  | Reported |
|  |  | chr9-69248006 C>G | c.2761-6C>G |  | Splicing | R | PM2, PP3 | 0.00 |  |  | 0.59 | Novel |
|  |  | chr9-69251033 A>G | c.3085-2A>G |  | Splicing | LP | PVS1, PM2 | 0.000001 |  |  | 0.97 | Novel |
| ***UGT1A1*** | NM_000463.3 | chr2-233772267 A>AG | c.1312dup | p.(Glu438Glyfs*71) | Frameshift | LP | PVS1, PM2 | 0.00 |  |  |  | Novel |
|  |  | chr2-233761107 TTTG>T | c.824_826del | p.(Val275del) | Inframe del | LP | PM1, PM2, PM4 | 0.00 |  |  |  | Reported |
|  |  | chr2-233772405 G>A | c.1448G>A | p.(Trp483*) | Nonsense | LP | PVS1, PM2 | 0.00 |  |  |  | Novel |
|  |  | chr2-233760961 T>G | c.674T>G | p.(Val225Gly) | Missense | LP | PM1, PM2, PP2, PP5 | 0.0005 | 0.290 | 6.1 |  | Reported |
|  |  | chr2-233767076 G>A | c.907G>A | p.(Val303Met) | Missense | R | PM1, PM2, PP2 | 0.000008 | 0.434 | 26.4 |  | Reported |
| ***USP53*** | NM_001371395.1 | chr4-119248883 G>A | c.372+1G>A |  | Splicing | LP | PVS1, PM2 | 0.00 |  |  | 1.0 | Novel |
|  |  | chr4-119256345 ACT>A | c.475_476del | p.(Leu159Valfs*2) | Frameshift | LP | PVS1, PM2 | 0.000002 |  |  |  | Novel |
| ***VIPAS39*** | NM_001193315.2 | chr14-77433895 C>G | c.1126 G>C | p.(Ala376Pro) | Missense | R | PM2 | 0.00 | 0.536 | 29.1 |  | Novel |
| ***WDR19*** | NM_025132.4 | chr4-39205619 C>T | c.773C>T | p.(Ser258Phe) | Missense | R | PM2, PP3 | 0.00 | 0.795 | 26.4 |  | Novel |
|  |  | chr4-39205624 C>T | c.778C>T | p.(His260Tyr) | Missense | R | PM2 | 0.000009 | 0.465 | 19.1 |  | Novel |
| ***YARS1*** | NM_003680.4 | chr1-32791235 T>C | c.611A>G | p.(Tyr204Cys) | Missense | R | PM2, PP3 | 0.000002 | 0.601 | 32 |  | Reported |
| ***ZFYVE19*** | NM_001077268.2 | chr15-40809911 C>CA | c.513dup | p.(Arg172Thrfs*8) | Frameshift | LP | PVS1, PM2 | 0.00 |  |  |  | Novel |

The pathogenicity of deteceted variants was classified according to the American College of Medical Genetics and Genomics (ACMG) guidelines (Richards et al., 2015). Combined annotation-dependent depletion (CADD) and rare exome variant ensemble learner (REVEL) scores for missense variants were obtained from University of California, Santa Cruz (UCSC) website <https://genome.ucsc.edu/>, while SpliceAI scores for splicing variants were obtained from <https://spliceailookup.broadinstitute.org/>. All gene symbols and nomenclature were according to the [HUGO Gene Nomenclature Committee (HGNC)](https://www.genenames.org/) website <https://www.genenames.org/>. All variant nomenclature complies with the guidelines of the human genome variation society (HGVS) <http://varnomen.hgvs.org/>. The chosen gene transcript is the most biologically relevant transcript for each gene (MANE-Select transcript) based on the ensembl database <http://www.ensembl.org/index.html>. Allelic frequencies were derived from the gnomAD (genome aggregation database): <https://gnomad.broadinstitute.org/> according to gnomAD version v.4.1.0; HGVSC, base change; HGVSP, protein change; LP, likely pathogenic; MAF, minor allele frequency; P, pathogenic; VUS, variant of uncertain significance. ACMG scoring abbreviations: PVS1, Null variant in a gene where loss of function is a known mechanism of disease; PS1, Same amino acid change as an established pathogenic variant; PS2, De novo in a patient with phenotype consistency, no family history and both maternity and paternity are confirmed; PS3, Well-established functional studies show a deleterious effect; PM1, Non-truncating non-synonymous variant is located in a mutational hot spot and/or critical and well-established functional domain; PM2, Extremely low frequency in the gnomAD population database; PM3: For recessive disorders, detected in trans with a pathogenic variant, or in a homozygous or compound heterozygous state in affected cases; PM4, Protein coding length changes as a result of in frame variant, and this variant is not located in a repeat region; PM5, Different amino acid change as a known pathogenic variant; PP1, co-segregation in multiple family members; PP2, Missense variant in a gene with low rate of benign missense mutations and for which missense mutation is a common mechanism of a disease; PP3, For a missense or a splicing region variant, computational prediction tools unanimously support a deleterious effect on the gene; PP5, Reputable source recently reports variant as pathogenic, but the evidence is not available to the laboratory to perform an independent evaluation.
